# Aggressive Digital Papillary Adenocarcinoma of the Hand Presenting as a Felon

**DOI:** 10.1155/2017/6456342

**Published:** 2017-03-21

**Authors:** Justin R. Bryant, Preston Gardner, Matthew Yousif, John C. Pui, Raymond T. Hajjar, E. Aron Haass

**Affiliations:** ^1^Plastic Surgery Fellowship, Beaumont Hospital-Farmington Hills, Farmington Hills, MI, USA; ^2^Orthopedic Surgery Fellowship, Beaumont Hospital-Farmington Hills, Farmington Hills, MI, USA; ^3^Pathology, Beaumont Hospital-Farmington Hills, Farmington Hills, MI, USA

## Abstract

Aggressive digital papillary adenocarcinoma is a rare eccrine sweat gland malignancy that is frequently misdiagnosed at initial presentation. Histologically, this tumor is similar in appearance to many adenocarcinomas and as such may be diagnosed as a metastatic lesion. We present the case of a patient with digital papillary adenocarcinoma, which was initially diagnosed as a felon. No consensus has been published regarding the treatment of this disease. A review of the diagnosis, pathology, treatment, and adjunctive treatments of aggressive digital papillary adenocarcinoma are also included.

## 1. Overview

Digital papillary adenocarcinoma is a rare malignancy of eccrine sweat gland origin with an acral distribution. Reports of aggressive digital papillary adenocarcinoma are predominately in middle-aged males and rarely in females [1, 2]. The typical presentation is of a solitary cutaneous nodule, which may or may not be painful, developing over the preceding weeks to years [2]. The most commonly reported location of occurrence has been the volar surface of distal digits of the hand, but occurrences elsewhere on the hand and feet have been reported. These lesions may invade into soft tissue, bone, and blood vessels locally and have a propensity for metastasis.

The entity “aggressive digital papillary adenoma” was initially described in 1979 by Helwig at the Armed Force Institute of Pathology (AFIP) who later differentiated between “aggressive digital papillary adenoma” and “aggressive digital papillary adenocarcinoma” in a 1987 case series [1, 3]. Histologic features such as cytologic atypia, mitotic figures, glandular differentiation, and necrosis were initially used to differentiate between these two species [1]. The term “aggressive” was utilized due to the tumors' tendency for recurrence and metastasis. Duke et al. later reexamined the cases in this series as well as subsequent cases encompassing a larger case study [4]. He suggested reclassification of both entities as “aggressive digital papillary adenocarcinoma” in 2000 as none of the histologic parameters used to differentiate between the two entities were found to be predictive of recurrence or metastasis [5]. Removal of the nomenclature “aggressive” has been suggested by Suchak et al. in 2012 and Chen and Asgari in 2014 due to its classification as a low-grade malignant tumor [2, 5]. Variability in use of the term “aggressive digital papillary adenocarcinoma” and “digital papillary adenocarcinoma” persists in the literature today. While case reports can be found in the literature, the only large case series are those of Kao et al., Duke et al., and Suchak et al. [1, 2, 4]. Here, we report a case of digital papillary adenocarcinoma of the finger and review previous reports of this entity in the hand.

## 2. Case Report

An 87-year-old male presented to his primary care physician with complaint of edema of his left fourth digit distal phalanx, for which he was initially treated with oral antibiotics. Two weeks later the patient reported continued edema and the appearance of localized erythema when seen in the emergency department. Radiographs found no abnormalities in the distal phalanx and the patient was treated with a dose of intravenous antibiotics and a different oral antibiotic and was given instruction to follow up with a hand surgeon.

**Figure 1:**
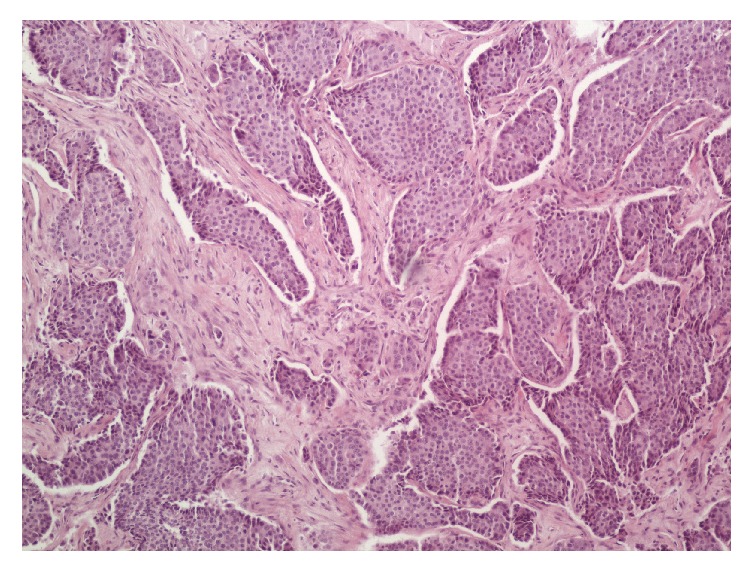
Lobules and cords of aggressive digital papillary adenocarcinoma (H & E, original magnification 100x).

**Figure 2:**
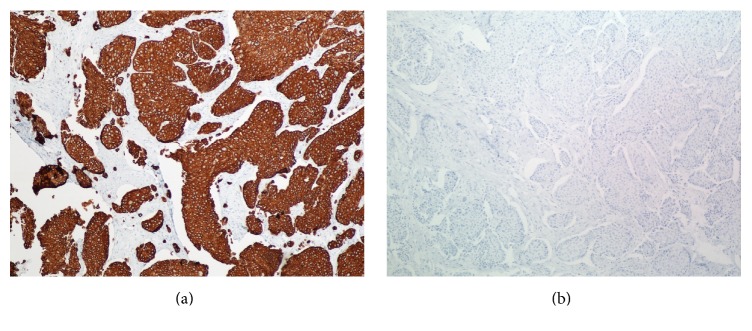
The carcinoma is positive for CK 7 (a) and negative for CK 20 (b) (H & E, original magnification 100x).

At the time of follow-up with the hand surgeon, the patient complained of pain in the fingertip in addition to the continued edema and erythema. This lesion appeared as a typical felon at the time of consultation; thus no photographs were obtained. Concern for felon led to incisional biopsy and irrigation. The histopathologic specimens from bone and soft tissue demonstrated small lobules, cords, and single epithelial cells set within a desmoplastic stroma (Figure 1). Immunohistochemical stains were strongly positive for cytokeratins CK 7 and CK AE1/3 but were negative for cytokeratins CK 20, TTF-1, prostate specific antigen, synaptophysin, chromogranin, p63, and S-100 (Figure 2). The histopathological differential diagnosis included a primary sweat gland carcinoma (aggressive digital papillary adenocarcinoma) as well as a metastatic adenocarcinoma from an upper gastrointestinal or pancreaticobiliary tract origin.

This diagnosis prompted ordering of an upper extremity MRI as well as computed tomography (CT) of the chest, abdomen, and pelvis, which revealed a 5.1 × 4.2 cm solid heterogenous right upper lobe hilar mass with irregular margins. An enlarged right axillary lymph node, multiple nonspecific mediastinal lymph nodes, and an enlarged right paratracheal lymph node were also noted. Of note, a normal chest radiograph had been performed one year prior to initial presentation.

**Figure 3:**
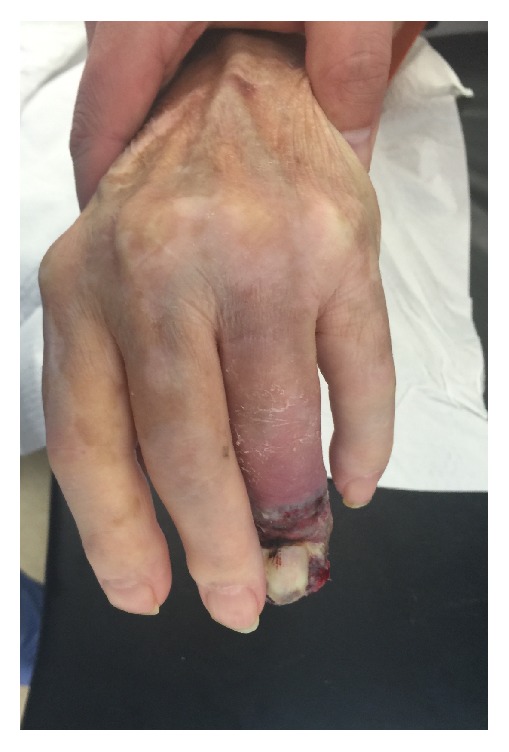
ADPA lesion after biopsy, dorsal view.

**Figure 4:**
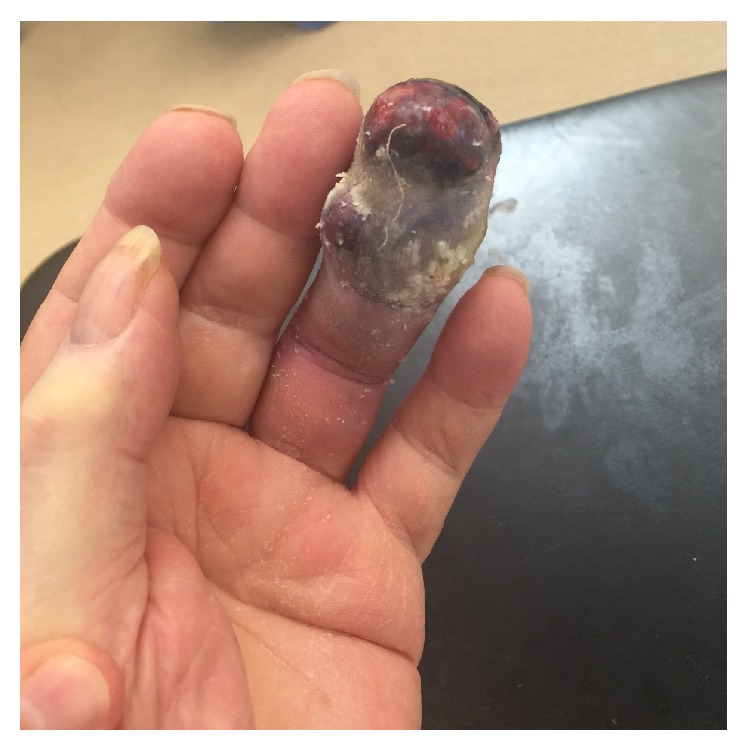
ADPA lesion after biopsy, palmar view.

**Figure 5:**
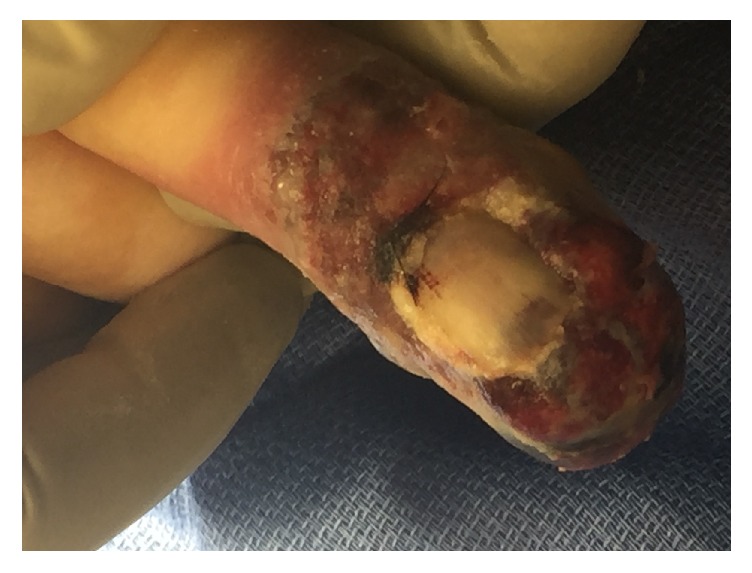
ADPA lesion prior to amputation, dorsal view.

**Figure 6:**
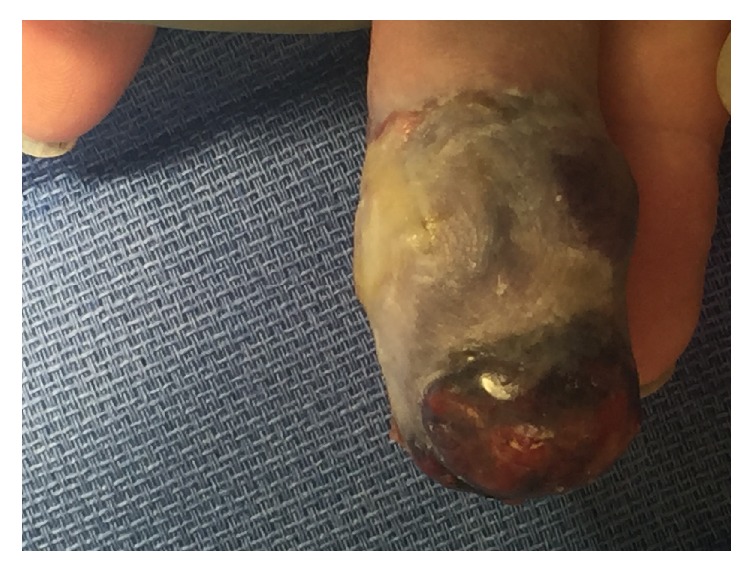
ADPA lesion prior to amputation, palmar view.

At the time of postoperative examination the finger had worsened in appearance with increased edema, ecchymosis, and pain (Figures 3 and 4). After discussion between the patient and the hand surgery team, the decision was made to proceed with amputation at the proximal phalanx one month after biopsy. Further progression in appearance at the time of scheduled amputation was observed (Figures 5 and 6). The amputation was tolerated well. Because of the patient's advanced age and location of the pulmonary hilar mass, the patient and family elected not to undergo biopsy of the pulmonary mass.

**Figure 7:**
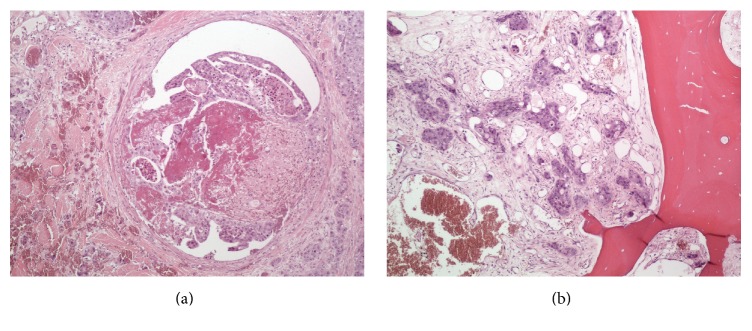
In the amputation specimen, the ADPA was found to be present within vascular spaces (a) as well as within the bone (b) (H & E, original magnification 100x).

Pathologic examination of the amputation specimen demonstrated an ulcerated poorly differentiated adenocarcinoma measuring 4.6 cm with involvement of the dermis, subcutis, and bone and lymphovascular/perineural involvement (Figure 7). Skin, soft tissue, and bony surgical margins were found to be uninvolved. In the absence of another primary source, these findings were consistent with a papillary digital adenocarcinoma.

## 3. Diagnosis

The average tumor size of ADPA has been reported as 1.7–2.0 cm with a range of 0.4–4.3 cm and is described as firm in consistency [2, 4]. The initial differential diagnosis at the time of presentation frequently includes hematoma, pyogenic granuloma, paronychia, cyst, vascular malformation, giant cell tumor, glomus tumor, pyogenic granuloma, foreign body granuloma, and benign adnexal tumor [2]. As such, these lesions are typically treated with either excisional or punch biopsy, ultimately achieving the diagnosis by histopathologic examination. The use of fine needle aspiration (FNA) has also been reported in establishing an early diagnosis of suspicious lesions or in evaluation of possible recurrence at sites of previous excision [6].

## 4. Histopathology

Histologically, these tumors are found to be multinodular and solid but may have cystic components with papillary projections as well [2, 4]. The tumor originates within the dermis, but extension into the subcutaneous tissue may be present as well as epidermal involvement [2]. Tubuloalveolar and ductal architectural patterns as well as focal infiltration are commonly reported [2, 4, 7]. Variable cytologic atypia has been reported, but these and other histologic parameters have been shown to be poor predictors of metastatic potential in this tumor. Because of this it has been suggested that this entity be regarded as “malignant with definite metastatic potential” [2, 4, 8]. A variety of histochemical and immunohistochemical stains have been used to differentiate from metastatic adenocarcinoma and other tumors. Dermatologic entities with similarities in histologic appearance include hidradenoma, apocrine cystadenoma, eccrine acrospiroma, papillary eccrine adenoma, apocrine adenoma, chondroid syringoma, and tubular apocrine adenoma [2, 4, 7]. Specimens from these tumors may also be misdiagnosed as metastatic disease due to similarity in histologic architecture to adenocarcinoma of the breast, thyroid, and gastrointestinal tract [2]. The biopsy specimen size is important as this entity may easily be misdiagnosed in small specimens [2]. Recently it was suggested that a histopathologic diagnosis of apocrine hidrocystoma or cystadenoma on an incisional biopsy specimen from the fingers or toes should raise the suspicion of possible underlying digital papillary adenocarcinoma [9].

## 5. Treatment

Initial excisional biopsy may be the first source of diagnosis but treatment has been the source of debate in the literature with no guidelines on surgical margins having been published. After diagnosis, reexcision with clear margins has been advocated in order to avoid local recurrence and metastasis [4]. Reports of Mohs micrographic surgery for excision after punch biopsy [2] and as a reexcision procedure [1] have been published but are few in number. In the case of ADPA of the digit, reexcision may be accomplished by either wide local excision (WLE) or partial digit amputation. For tumors of the distal phalanx, clear margins may be achieved by distal phalanx amputation. Elsewhere in the hand specific margins for wide local excision they have not been agreed upon or recommended.

Duke's review of 67 cases from the AFIP consisted of 85% of lesions located on the hand (79% on fingers) [4]. After reclassifying this entity as not having a benign counterpart, a more aggressive approach was taken with an increased rate of reexcision or amputation after diagnosis. To avoid local recurrence or metastasis, Duke et al. [4] recommended complete neoplasm excision as a secondary procedure after diagnosis.

Hsu et al. [10] completed a systematic evaluation of the literature examining excision compared to amputation in the treatment of ADPA. Though this study included lesions of the foot and groin, 58% of the 19 reviewed cases were of occurrence in the fingers. The authors found no difference in clinical data and prognosis between patients who underwent WLE or amputation and recommended consideration of WLE with or without sentinel lymph node biopsy instead of amputation in cases of long standing ADPA without metastasis or bony invasion.

Suchak et al. [2] reported a retrospective clinicopathologic case series of 31 patients with ADPA, 84% of which were located in a finger. The patients in their study had been treated by either complete excision or digit amputation. Two of the patients who initially had complete excision later had recurrence and were treated with digit amputation. Their recommendations were for either aggressive reexcision or conservative partial amputation with a result of clear surgical margins.

## 6. Recurrence

The reported overall recurrence rate in patients diagnosed with ADPA is 21–28% [2, 4] with lower rates in those undergoing subsequent reexcision or amputation after receiving diagnosis from their initial excision than in those without further surgical excision [2, 4]. Patients who are not treated by reexcision or amputation have a reported recurrence rate of 21–50% [2, 4] whereas those who are treated with either reexcision or amputation have a reported recurrence rated of up to 5% [4].

## 7. Sentinel Lymph Node Biopsy

Malafa et al. [11] likened ADPA to melanoma in its predilection for metastasis and adapted the concept of sentinel lymph node biopsy (SLN) in the treatment of aggressive digital papillary adenocarcinoma in 2000. However, only a few case series have been reported in regard to SLN biopsies. Bogner et al. [12] described the use of SLN biopsy in sweat gland carcinoma including two patients with ADPA of the finger. Positive SLN biopsy led to regional lymphadenectomy, with no further lymph node metastasis found. They advocated the use of SLN biopsy for detection of subclinical metastasis in these tumors. Duke et al. reported use of SLN biopsy in four of their patients undergoing digit amputation, all of which resulted as negative and believed it was premature to advocate routine SLN biopsy [4]. Although no formal studies have been undertaken regarding the utility of lymphatic mapping and SLN biopsy in aggressive digital papillary adenocarcinoma, there have been several case reports. These have reported the use of SLN biopsy for detection of metastasis but have also recognized the need for further studies to evaluate the impact and necessity while recognizing that rarity of this tumor makes clinical studies difficult [12, 13]. At present there are no specific recommendations regarding indications for SLN biopsies in ADPA patients and no significant evidence for improving long-term survival.

## 8. Metastasis

The reported overall rate of metastatic disease for patients diagnosed with ADPA is 14–26% and may occur with or without local recurrence [2, 4]. Metastases may be seen in regional lymph nodes and the most commonly reported site of distant metastasis is the lung. Patients may initially present with metastasis in addition to their primary tumor, though distant disease is rare at initial diagnosis [2, 4]. Regional lymph node metastasis at the time of initial presentation has been reported at 3–5%, though the duration of primary tumor before presentation was not reported [2, 4]. Aggressive surgical treatment of limited localized metastatic disease has been advocated including lobectomy for pulmonary metastases [4]. Patients with pulmonary metastasis have been reported as alive and well without evidence of disease as long as 13 years after lobectomy [4].

## 9. Chemotherapy

The use of adjunctive systemic therapy has been reported in few cases for the treatment of pulmonary metastases after initial treatment [1, 4, 7, 14]. Frey et al. reported some initial decrease in pulmonary metastatic lesion size with carboplatin and paclitaxel, but due to the development of neuropathy the chemotherapeutic regimen was changed with ensuant lesion size increase and death [7]. Ultimately all of the cases reported little or no improvement with no obvious benefit. No standardization of the use of adjunctive chemotherapy or regimens have been agreed upon, but agents reported include cisplatin, gemcitabine, 5-FU, mitomycin, adriamycin, thiotepa, fluoxymesterone, carboplatin, paclitaxel, docetaxel, and VP-16 [1, 4, 7, 14].

## 10. Radiation Therapy

Adjunctive radiation therapy has only been reported in one case report where it was used to reduce tumor size preoperatively in a ADPA lesion of the hand [15]. The authors reported complete clinical and histologic tumor regression without evidence of recurrence or metastasis at 6-month follow-up. Although the authors advocated preoperative radiation therapy to decrease tumor size, no consensus or further studies have been published.

## 11. Importance of Follow-Up/Monitoring

The propensity for recurrence and metastasis in ADPA warrant staging when a diagnosis is made including a thorough physical examination and radiologic studies. As metastatic disease has been reported as late as 20 years after definitive excision [4], long-term follow-up is also important.

## 12. Conclusion

Here, we discussed the presentation of a single patient with ADPA complimented by a review of the literature. There remains a paucity of literature on the disease process and treatment approaches, particularly in regard to advanced modalities such as sentinel lymph nodes, chemotherapy, and radiation. As with all scientific topics, prospective randomized trials would give stronger direction for ADPA; however due to the rarity of the disease process, it will remain difficult to perform a large cohort trial.
